# County level study of the interaction effect of PM_2.5_ and climate sustainability on mortality in China

**DOI:** 10.3389/fpubh.2022.1036272

**Published:** 2023-01-06

**Authors:** Yanan Guo, Linsheng Yang, Hairong Li, Leijie Qiu, Li Wang, Lantian Zhang

**Affiliations:** ^1^Key Laboratory of Land Surface Pattern and Simulation, Institute of Geographical Sciences and Natural Resources Research, Chinese Academy of Sciences, Beijing, China; ^2^College of Resources and Environment, University of Chinese Academy of Sciences, Beijing, China

**Keywords:** PM_2.5_, climate sustainability, mortality, China, interaction effect

## Abstract

**Introduction:**

PM_2.5_ and climate change are two major public health concerns, with majority of the research on their interaction focused on the synergistic effect, particularly for extreme events such as hot or cold temperatures. The climate sustainability index (CLS) was introduced to comprehensively explore the impact of climate change and the interactive effect on human health with air pollution.

**Methods:**

In this study, a county-level panel data in China was collected and used. The generalized additive model (GAM) and geographically and temporally weighted regression (GTWR) was used to explore the interactive and spatial effect on mortality between CLS and PM_2.5_.

**Results and discussions:**

Individually, when CLS is higher than 150 or lower than 50, the mortality is higher. Moreover, when PM_2.5_ is more than 35 μg/m^3^, the influence on mortality is significantly increased as PM_2.5_ concentration rises; when PM_2.5_ is above 70 μg/m^3^, the trend is sharp. A nonlinear antagonistic effect between CLS and PM_2.5_ was found in this study, proving that the combined adverse health effects of climate change and air pollution, especially when CLS was lower (below 100) and PM_2.5_ was higher (above 35 μg/m^3^), the antagonistic effect was much stronger. From a spatial perspective, the impact of CLS and PM_2.5_ on mortality varies in different geographical regions. A negative and positive influence of CLS and PM_2.5_ was found in east China, especially in the northeastern and northern regions, -which were heavily polluted. This study illustrated that climate sustainability, at certain level, could mitigate the adverse health influence of air pollution, and provided a new perspective on health risk mitigation from pollution reduction and climate adaptation.

## 1. Introduction

Particles measuring less than 2.5 [(PM_2.5_) micrometers in aerodynamic diameter] is the most representative and harmful air pollutant, and has received increased attention in recent years. Scientists have reached a consensus that long-term exposure to air pollution contributes to an increased risk of illness and premature death from ischemic heart disease, lung cancer, chronic obstructive pulmonary disease (COPD), lower-respiratory infections (e.g., pneumonia), stroke, type 2 diabetes, and, more recently, adverse birth outcomes (1–7). PM_2.5_, carrying harmful chemicals, can penetrate deeply into the human lung system and blood circulation to cause cardiovascular and respiratory diseases, resulting in an estimated 42 million deaths (~7.6% of the total mortality) and 1031 million disability-adjusted life-years lost (8). A positive correlation between PM_2.5_ and mortality is proven (9–11). For instance, Yap et al. found that exposure to particulate air pollution was significantly associated with non-accidental mortality and cardiovascular mortality, especially in the elderly ≥65 years (12).

Studies on the health effects of climate change mostly focus on the effect of high temperature or heat waves (13–15). There are few studies on the health effects of extremely low temperature, as many scientists believe that it has less impact than extreme high temperature (16–18). Moreover, few studies have been carried out on the impact of diurnal temperature on health (19). Longden noted that the reference values used to define extreme temperatures (high and low temperature) under different climatic zones in Australia influenced the estimation of the impact of death, reversing the previous view that extreme low temperature was the main cause of death in Australia, and thus proposed the net benefit of the health impact of climate change (20). The influence of climate change on health may vary from one region to another, for example, the mortality from climate-induced conditions was increased in Brasilia and decreased in the Russian subarctic (21, 22). In China, a study found that the mortality resulting from extremely high temperatures was higher in the north than in the south, while the mortality from extremely low temperatures was higher in the south (23), implying the climate adaptability of the locals. Most of the research on climate and mortality are based on temperature, and the relationship of mortality with other factors like wind, pressure or the composite climate index are lacking. The reductionist approach of merely using air temperature in assessing weather-health relationships, specifically hot, or cold extremes, can limit our understanding of human-weather interactions (24).

Air quality is closely related to meteorological conditions. Studies have shown that static weather, extremely high temperatures, and extreme composite weather can increase the concentration of ozone and particulate matter in the air (18). Due to the strong correlation between air quality and climatic conditions (25), there have been studies on the synergistic health effects of the two in recent years, mainly through the establishment of indicators like relative excess risk due to interaction (RERI). Borge et al. found that the weather change has made air-quality related-mortality up to 10% greater in Spain for the period (1993–2017) (26). Ho et al. (27) found that areas with higher PM_2.5_ of lag two day of a hot hazy day had a significantly higher all-cause mortality [odds ratio (OR): 1.135], and on a cold hazy day, the OR was 1.131 with the lag of 0 days (27). Many studies suggest that future warming may increase PM_2.5_-related deaths, and the health benefits of climate policy may be offset by severe climate-induced PM_2.5_ and aging (28). Moreover, considering the physiological stress that extreme weather and pollution may cause, Zhang et al. established the human thermal comfort index combining air pollution and somatosensory temperature (29). In summary, the impacts of air pollution and climate on health are mostly based on the negative effects of climate factors (most are single indicators), while few studies explain the relationship between them from the perspective of population adaptability (climate sustainability).

China spans five climatic zones, having the world's largest altitude range, with a varied climatic condition throughout the country. China also experienced severe air pollution in the past decades. Since its accession to the World Trade Organization in 2001, the share of heavy & chemical industries such as energy, steel, and chemical engineering increased sharply. The total energy and materials consumption increased after 2000, and the urban residential energy consumption increased as the population increased (30, 31). From 1995 to 2010, the concentration of PM_2.5_ showed a fluctuating upward trend of approximately 7.6 μg/m^3^ 5 years, and the bulk of this increase occurred from 2000 to 2010 (32).

Therefore, in order to explore the interactive effect of climate adaptability and air pollution, this study established a climate sustainability index (CLS) and explored the interactive and spatial effect on mortality between CLS and PM_2.5_ based on the county-level death in 2000 and 2010 in China, using GAM (Generalized additive model) and GTWR (Geographically and Temporally Weighted Regression) sequentially. This study can provide scientific support for addressing the health risks of air pollution and adapting to climate change in the meantime.

## 2. Methods and data

### 2.1. Method

A two-stage analytic approach was used in this study. In the first stage, GAM is built. PM_2.5_ and CLS independently, and the interaction of PM_2.5_ and CLS together with socioeconomic confounders are introduced sequentially in the model. The mortality panel data consists of 2000 and 2010, and temporal variations of mortality are set as the dependent variables, respectively, in GAM. In this stage, the interaction between PM_2.5_ and CLS, and the confounding effect of socioeconomic conditions are explored. In the second stage, a GTWR is introduced. Again, the mortality panel data in 2000 and 2010, and temporal variations of mortality are set as the dependent variables, and all of the independent variables but not the co-founders with no significance are included in GTWR. In this stage, the model spatially varying relationships between PM_2.5_, CLS, and total mortality are explored. For GAM, the result is expressed as the spin curve of exp (β), and for GTWR, the result is expressed as the exp (β), representing the OR of PM_2.5_ or CLS on mortality.

#### 2.1.1. Global and local Morans'I

Global Moran's I, which is introduced in 1948, is extensively used to test the spatial autocorrelation based on feature locations and attribute values. This study employs Moran's index to explore the clustering effect of mortality. The calculation method for Moran's I in this study referred from Anselin (33).

#### 2.1.2. Gini index

Gini index is a conventional inequality measure, and has been widely used in income and health related studies (34). It assumes that transfers at the top of the distribution (e.g.. a transfer from the richest person to the second richest person) reduce inequality as much as if the same transfers happen at the bottom of the distribution. In this context, Gini index is used to represent the inequity of mortality inside a province and inside China, using the county level data. The absolute Gini index is calculated using the Lorenz curve (35, 36), based on the total mortality of each county ranked from the worst to the best level using the following formula,


$$\begin{array}{l} G=2\overset{T}{\underset{t-1}{\sum }}u_{t}\times f_{t}\times R_{t}-u\; \end{array}$$

Here, G is the Gini index, u is the mean value of the total mortality, T is the number of counties, f is the weighed share, here we use constant number 1 as the mortality instead of mortality. R is the relative rank of the t^th^ county.

#### 2.1.3. GAM

GAM is widely used for time-series analysis on the impact of air pollution and/or climate factors on health (37, 38). It allows for non-parametric adjustments for nonlinear confounding effects. In this study, firstly, a GAM with quasi-Poisson regression was performed to obtain the risk estimates of mortality due to PM_2.5_ and CLS separately with penalized smoothing spline function(s), other factors, including education, economic etc. in Table 1 were also included as confounders. The main model is:


$$\begin{array}{l} f\left(x\right)=s\left(PM_{2.5},\;df\right)+s\left(CLS,\;df\right)+\overset{n}{\underset{i}{\sum }}S\left(cof_{i},\;df\right)\; \end{array}$$

f(x) here represents estimated number of mortality in each county; df represents the degrees of freedom. After model test to avoid over fitting based on the value of vif, the df for PM_2.5_ and CLS were set as 4 and 5 respectively, and 4 df for all the other confounders.

**Table 1:** Variable definitions and summary statistics.

	**2000**				**2010**				**2010–2000**			
	**Mean**	**Max**	**Min**	**Std**	**Mean**	**Max**	**Min**	**Std**	**Mean**	**Max**	**Min**	**Std**
Total mortality (‰)	6.01	12.24	0.57	1.45	5.73	12.81	0.75	1.56	−0.28	3.89	−7.92	1.19
PMP (%)	16.13	99.78	0	28.88	16.19	99.78	0	28.90	−54.18	54.65	−5.17	3.08
MED (‰)	19.90	139.72	1.26	12.94	26.19	135.25	1.77	14.69	6.29	86.90	−59.37	9.51
EDU (Year)	7.40	11.71	0.63	1.47	8.69	13.14	2.00	1.43	1.29	3.98	−1.47	0.45
ELD (%)	6.74	18.15	0.97	1.72	8.79	19.00	1.02	2.22	2.04	8.61	−5.12	1.44
GDPpc (1000 yuan)	5.19	34.80	0.83	3.07	23.48	240.40	2.79	17.63	18.29	229.53	−10.07	16.51
PM_2.5_ (μg/m^3^)	38.04	89.41	1.76	14.94	47.36	129.35	1.15	20.10	9.32	52.21	−10.50	7.03
CLS	116.75	253.15	−183.19	63.87	119.39	251.83	−179.30	64.01	2.63	34.95	−20.24	5.83

Secondly, to include the interaction effect, a modified GAM was used as following,


$$\begin{array}{l} f\left(x\right)=s\left(PM_{2.5},\;df\right)+s\left(CLS,\;df\right)+ti\left(PM_{2.5},\;CLS\right)\\ \;+\overset{n}{\underset{i}{\sum }}S\left(cof_{i},\;df\right) \end{array}$$

where, function (ti) here is introduced to process the interaction between smooths of PM_2.5_ and CLS. The “mgcv” package was used to process the above modellings in R 4.2.0.

#### 2.1.4. GTWR

GTWR is a method of regression analysis proposed by Huang et al. (39) considering the simultaneous resolution of space and time characteristics of data. GTWR assumes that the regression coefficient is an arbitrary function of the geographical location and the time of an observation considering the non-stationarity of time and space. The GTWR principle is as follows (39):


$$\begin{array}{l} y_{i}=\beta_{0}\left(u_{i},v_{i},t_{i}\right)+\overset{d}{\underset{k=1}{\sum }}\beta_{k}\left(\mu_{i},v_{i},t_{i}\right)x_{ik}+\varepsilon_{i} \end{array}$$

Where, (yi, xi_1_, xi_2_,^...^xi_d_) are the n sets of observations at the observation point (u_i_, v_i_, t_i_), the dependent variable y_i_ is the total mortality in this study, and the independent variables x_i_ represent PM_2.5_, CLS and other covariates which are shown in Table 1. β_0_ (u_i_, v_i_, t_i_) is the intercept value. β_k_ (u_i_, v_i_, t_i_) is a function of coefficients, it's the value of the function β_k_ (u, v, t) at the ith observation point (u_i_, v_i_, t_i_). The estimation of β_k_ (u_i_, v_i_, t_i_) based on local weighted least squares, and it can be expressed as follows:


$$\begin{array}{l} \hat{\beta }\left(u_{i},v_{i},t_{i}\right)={\left[X^{T}W\left(u_{i},v_{i},t_{i}\right)X\right]}^{-1}X^{T}W\left(u_{i},v_{i},t_{i}\right)Y \end{array}$$

The weight *W*(*u*_*i*_, *v*_*i*_, *t*_*i*_)is the distance function from observation point (ui, vi, ti) to other observation points, usually using Gaussian distance function. In addition, the broadband selection has a great impact on the accuracy of the GTWR model, according Huang et al. (39) the AIC criterion can be used, that is, the minimum AIC corresponds to the optimal broadband.

### 2.2. Data source and process

#### 2.2.1. Mortality data

Census-based data at various spatial levels is popularly used in pollution-related epidemiology or ecological studies (1, 11). It can represent the health status at the population level. The data used in this study covers the total mortality data at county levels in 2000 and 2010 based on the 5th and 6th national censuses. It is calculated through the ratio of the total mortality to the total population from all the age groups, represented as thousandths (‰). There are 2,858 counties in China, and after data validation, 2790 counties with mortality remained. The abnormal values detected at the significant level of α = 0.01 are regarded as highly abnormal and are eliminated.

#### 2.2.2. Data of PM_2.5_ and CLS

Annual data of PM_2.5_ are derived from the V4.CH.03 product (China Regional Estimates) developed by the Washington University Atmospheric Composition Analysis Group (40, 41), and the mean value of PM_2.5_ was calculated by county administrative division. Spatiotemporal PM_2.5_ concentrations were primarily estimated utilizing the GEOS-Chem chemical transport model by combining aerosol optical depth (AOD). These modeling estimates were subsequently calibrated to regional ground-based PM_2.5_ observations using GWR (41). This well-developed space–time model exhibited excellent performance in predicting annual mean geophysical PM_2.5_ estimates, showing a high consistency (*R*^2^ = 0.81) with globally distributed ground monitors. GWR is applied to account for PM_2.5_ residual with ground monitors yielding a cross-validation value of *R*^2^ = 0.90–0.92 (40). The data are distributed as Geotiff files, and we use a mask tool to extract them at the county level. The annual values were summarized by zonal statistics tools using ArcMap 10.8.

Climate sustainability represents the people's somatosensory of the climate environment. In this study, CLS integrating climate factors and altitude adaptability is established based on Zhong et al. (42). The temperature-humidity Index, wind-chill index, clothing index, and altitude adaptation index were established first and then constructed into a comprehensive index with weighted coefficients, and the detailed calculation are shown in the Supplementary material. The meteorological data was obtained from the China Meteorological Data Service Center, and the 30 m DEM data is from the resdc (https://data.cma.cn/).

Compared with previous studies on the impact of climate change and environmental pollution on health, CLS can comprehensively reflect the human body's impact on the change of basic climate factors such as temperature, humidity and altitude, and provide a new perspective to analyze the impact of climate change on human health, so as to arouse more comprehensive impacts of changes in various climate conditions on human health except for extreme temperature.

#### 2.2.3. Other socio-economic data

Considering that 2000–2010 was the period of rapid economic development in China, the improvement of relevant socio-economic and medical conditions could affect the change in mortality. Furthermore, the number of ethnic minorities was also one of the important factors affecting the mortality in China at that time (43, 44). Therefore, this study incorporates the considerations of economic development, education level, medical resources and in national and local county levels, which are characterized by GDP per capita (GDPpc), years of education (EDU), medical beds per 1000 (MED), and the proportion of minority population (PMP) (44–46). Furthermore, numerous studies have shown that elderly people are sensitive to climate change and air pollution (4, 47). An aging population will aggravate health losses caused by climate change and air pollution. Thus, we considered the proportion of people aged 65 years and above (age≥65) (ELD) as a correction factor. All the data came from the national censuses, the China County Statistical Yearbook and China Urban Statistical Yearbook.

## 3. Results

### 3.1. Spatial distribution patterns of mortality and mortality change

The total mortality at the national level was 5.92 and 5.58 in 2000 and 2010, respectively. At the county level, the average total mortality was 6.20 ranging from 0.89 to 12.24 for 2000, and 5.93, ranging from 0.78 to 11.74 for 2010, respectively (Table 1). The total mortality showed a spatial cluster pattern for both years. In 2000 and 2010, the global Moran's I was 0.225 with highly significant. More specifically, in 2000, the southwest regions, including Sichuan, Tibet, Yunnan, and Guizhou provinces, and south Qinghai provinces had a higher mortality of above 6. The northeast and northwest regions, mainly Xinjiang, Inner Mongolia, Heilongjiang, and Jilin provinces had a lower mortality below 6. Counties surrounding Beijing and Tianjin, Shanghai, and the counties in the Pearl River estuary, had considerably higher mortality. In 2010, compared with 2000, the mortality distribution changed. Regions with high mortality <6 in Southwest China were relatively reduced, while regions in the eastern coastal region had increased, mainly in Jiangsu, Anhui, and Shandong provinces. Simultaneously, the areas with low mortality in the northwest and northeast also increased relatively, mainly in Gansu and Qinghai provinces. The county-level total mortality in China experienced a significant decrease from 2000 to 2010 (Figure S1).

**Figure 1:**
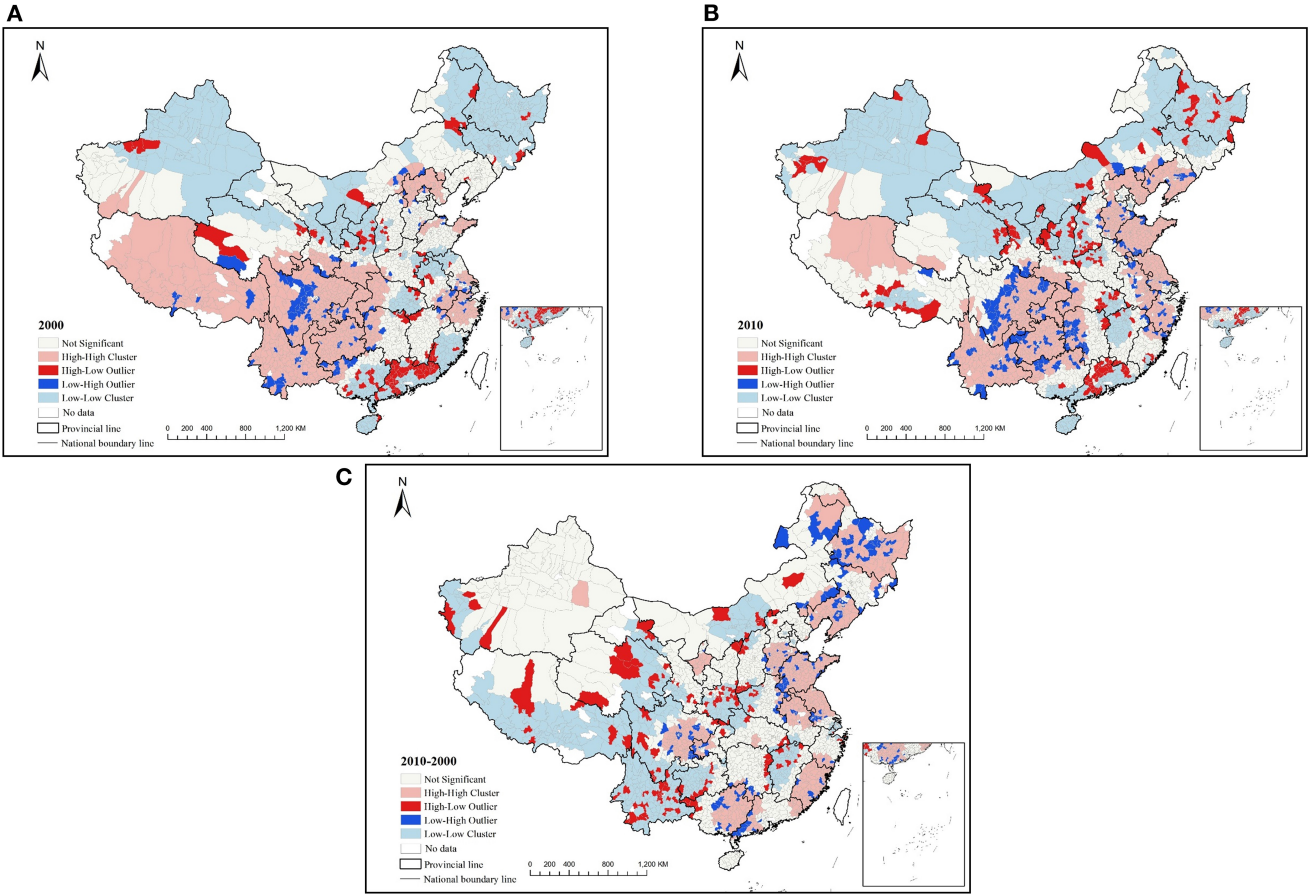
LISA clustering of mortality and mortality change **(A)** 2000 **(B)** 2010 **(C)** 2010–2000.

Based on the LISA (Figures 1A, B), in 2000, the cold spot (low-low) was distributed in the northern Xinjiang, Ningxia, and its surrounding areas, Heilongjiang, Jilin, southern Guangxi, Fujian, Pearl River Delta, Hainan, and eastern Hubei. The hot spot (high-high) was mainly distributed in the northern Hebei, Pearl River Delta, and southwest regions, including Tibet, Sichuan, Yunnan, Guizhou, and Chongqing. In 2010, the cluster pattern was more continuous compared with 2000. The cluster counties in the northern regions (low-low) and middle coastal regions (high-high) had increased, and the cluster counties in the southeast coastal regions (low-low) and southwest regions (high-high) had decreased. The results were similar to the previous spatial heterogeneity analysis results at the city level (48).

The spatial autocorrelation of the mortality changes from 2000 to 2010 showed that the global Moran's I was 0.168 with significant, though the value was small, indicating that there was a certain spatial correction in the mortality change. However, the distribution of the cold and hot spots of the LISA was more dispersed (Figure 1C), such as the cold spot (low-low) mainly distributed in Tibet, Yunnan, Qinghai, Jiangxi, and Hubei provinces, and the hot spot (high-high) was mainly distributed in Guangxi, Fujian, Jiangsu, Anhui, Shandong, Liaoning, and Heilongjiang.

### 3.2. Inequities of mortality

**Figure 2:**
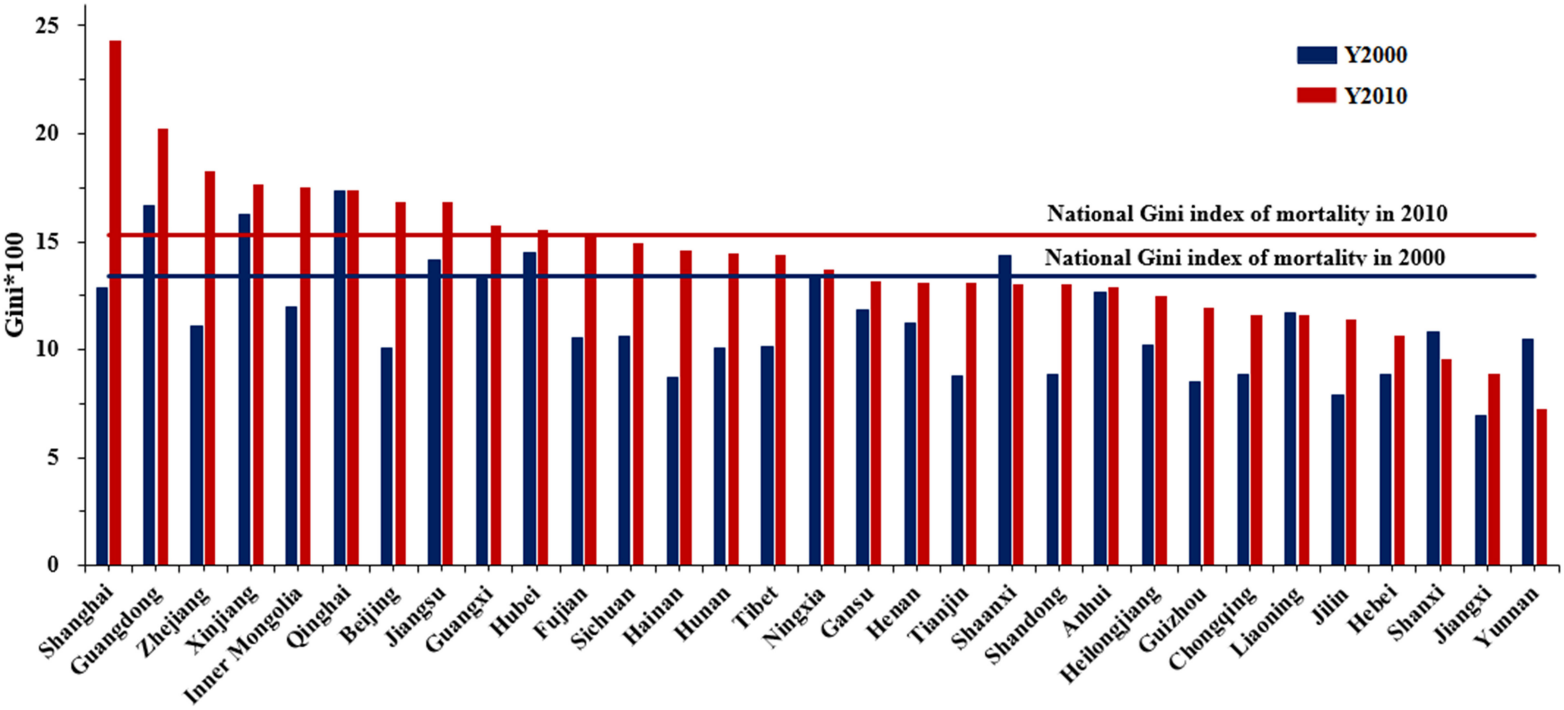
Gini index of mortality within provincial administrative unit for 2000 and 2010.

In 2000, the Gini indexes of mortality were higher in Qinghai, Guangdong, Xinjiang, Hubei, Shaanxi, and Jiangsu, and much lower in Jilin and Jiangxi. In 2010, Shanghai, Guangdong, Zhejiang, Xinjiang, Inner Mongolia, Qinghai, Beijing, Jiangsu, Guangxi, and Hubei had a higher Gini index, while for Jiangxi and Yunnan were considerably lower. Except for Shaanxi, Liaoning, Shanxi, and Yunnan, the Gini index of mortality of all provinces increased from 2000 to 2010, with a significant increase for Shanghai, Zhejiang, and Beijing. The Gini index (^*^100) of total mortality in China was 13.38 and 15.29 in 2000 and 2010, respectively (Figure 2). For both years, most of the Gini indexes at the provincial level were lower than the national index, indicating that there was considerable inequity among provinces. In all provinces, apart from Beijing and Guangdong in 2010, the Gini indexes were all below 0.2, indicating that the total mortality variations within provinces were smaller compared with the variation among provinces. Therefore, it is necessary to consider spatial heterogeneity to explore the impacting factors of mortality.

### 3.3. Influence of PM_2.5_ and CLS on mortality

**Figure 3:**
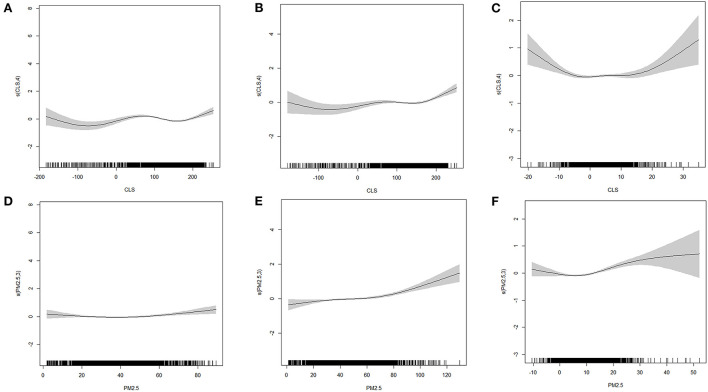
The GAM model fitting diagram of CLS, PM_2.5_ without interaction **(A, D)** 2000 **(B, E)** 2010 **(C, F)** 2010–2000.

Regression based on the cross-section data in 2000 and 2010 indicated that both CLS and PM_2.5_ had a significant influence on mortality. The change curves of CLS and PM_2.5_ tended to be similar in both years. For CLS (Figures 3A, B), the change is grouped into four states. From −200 to −50, the influence of CLS showed a significant decline, from −50 to 75 and more than 150, the influence was increased. The phase of 75 to 150 was slightly different; it was steady in 2010 but decreased in 2000. For PM_2.5_ (Figures 3D, E), its impact on mortality was significantly positively correlated. The change was more steady in 2000, while in 2010, it was generally stable below 60 μg/m^3^, and then showed a significant upward trend.

Based on the regression of changes in the 2 years (Figures 3C, F), the influence of CLS and PM_2.5_ on mortality was significant. For CLS, its effect change curve on mortality was similar to an inverted u, it decreased from −20 to −5 and increased at <15. For PM_2.5_, the effect showed an upward trend when the concentration change was >10μg/m^3^.

### 3.4. Interactive effect of PM_2.5_ and CLS on mortality

**Figure 4:**
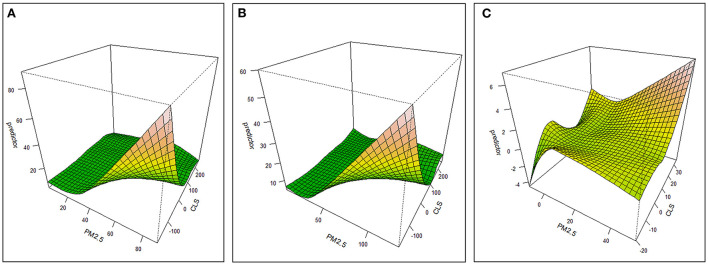
Three-dimensional effect graph of PM_2.5_-CLS interaction on the variation of mortality **(A)** 2000 **(B)** 2010 **(C)** 2010–2000.

In the GAM results, the degree-of-freedom (DOF) was >1, and the DOF of interaction factors was more than 10. It showed that the effect of interaction between CLS and PM_2.5_ on mortality was non-linear. The parameters in Table 2 showed that the interaction improved the interpretation of this model, and the influence of interaction factors on mortality and its change were significant at the *p* = 0.01 level. From the interaction point of view, the higher the PM_2.5_ concentration and the lower the CLS, the greatest the interaction effect on mortality (Figure 4). In both years, PM_2.5_ began to show an inflection point at about 30 μg/m^3^. When PM_2.5_ was <30 μg/m^3^, the overall interaction changed slightly, and when PM_2.5_ was >30 μg/m^3^, there was a significant impact. The inflection point of CLS appeared in 90–100, and when less than this range, the interaction increased significantly. However, when the CLS was between 100 and 200, the change of interaction was not obvious in 2000, while increasing in 2010, and when CLS exceeded 200, the interaction also decreased significantly, indicating that at this stage, the leading role of climate sustainability was stronger.

**Table 2:** The parameters for GAM in two periods with interactions.

	**f0**	**f1**	**f10**	**f0in**	**f1in**	**F2in**
Intercept	6.01	5.730	−0.280	6.398	5.982	−0.273
CLS (*p-*value)	0	0	0	0	0	0
PM_2.5_ (*p*-value)	0.002	0	0	0	0	0
In (CLS, PM_2.5_) (*p-*value)	/	/	/	0	0	0.005
PMP (*p-*value)	0	0.002	0.003	0	0	0.002
MED (*p-*value)	0	0	0.039	0	0	0.023
EDU (*p-*value)	0	0	0	0	0	0
GDPpc (*p-*value)	0	0.610	0	0	0.634	0
ELD (*p*-value)	0	0	0	0	0	0
Intercept P	0.00	0	0	0	0	0
*R*^2^(adj)	0.616	0.609	0.270	0.643	0.628	0.275
Deviance explained	61.9%	61.2%	27.6%	64.8%	63.3%	28.4%

f0, f1, f10 mean GAM results of 2000, 2010 and 2010-2000 without interactions; f0in, f1in, F2in mean GAM results with interactions.

This also showed that when the PM_2.5_ was below 30–35 μg/m^3^, even if CLS is very low, the interaction between the two had a low effect on health. When the CLS was between 100 and 200, the improvement of PM_2.5_ had a slight impact on mortality. When the CLS was >200, even if the PM_2.5_ concentration is high, the interaction between the two had a very low impact on mortality. Therefore, at this stage of >200, CLS began to play a dominant role.

**Figure 5:**
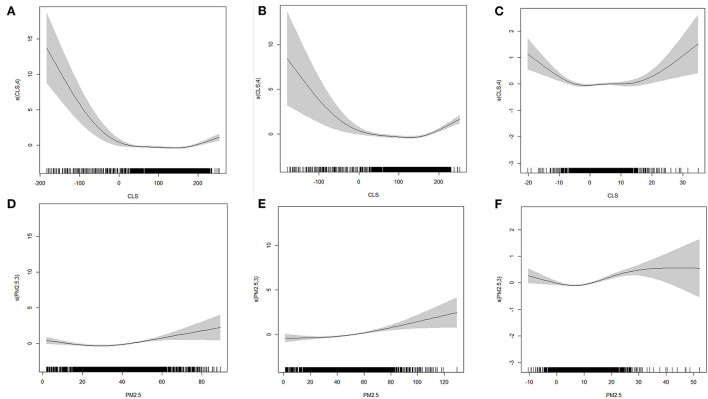
The GAM model fitting diagram of CLS, PM_2.5_ with interaction **(A, D)** 2000 **(B, E)** 2010 **(C, F)** 2010–2000.

After considering the interaction of air pollution and climate sustainability, the consistency of the impact trend of PM_2.5_ and CLS on mortality was enhanced, and the curve change was smoother (Figure 5). When PM_2.5_ was more than 35–40 μg/m^3^, its impact on mortality suggested an increasing trend in 2000, and significantly increasing trend in 2010. While the impact was not that obvious below this value in both 2000 and 2010. This indicated the impact of PM_2.5_ on mortality in 2010 was more remarkable than in 2000. For climate sustainability, when CLS was <50, the overall impact on mortality was significantly positive. The worse the CLS was, the higher the mortality. Moreover, between 100 and 150, the impact was not obvious. When CLS was more than 150, a positive correction was found. The CLS was mainly distributed in the Northeast areas (Figure S2), where the high chill index may be uncomfortable for people, thus causing an increase in mortality.

### 3.5. Spatial-temporal differentiation mechanism of PM_2.5_ and CLS on mortality

The regression results of the TWR, GWR and GTWR models were presented in Table 3. The AIC value of the GTWR model (14403.9) was much lower than that of the TWR model (15490.6) and GWR model (14570.1), representing that the GTWR model is the most suitable to use. The *R*^2^ of the GTWR model (0.675) was higher than that of the TWR (0.593) and GWR model (0.660), which means the highest explanatory power of GTWR.

**Table 3:** Comparison of the model test results.

	**TWR**	**GWR**	**GTWR**
*R* ^2^	0.593	0.660	0.675
Adjusted *R*^2^	0.593	0.660	0.674
Residual sum of squares	5212.74	4353.40	4172.46
AICc	15490.6	14570.1	14403.9
Bandwidth	0.206	0.115	0.114

Table 4 indicated that PM_2.5_ had a positive driving effect on mortality. In terms of the mean value, the risk of total mortality increased by 0.46 for every 100 μg/m^3^ increase of PM_2.5_. There was a negative correlation between CLS and mortality, with an increase of 100 of CLS, the total mortality decreased by 0.10. Moreover, it can be observed from the table that increased economic and educational levels contribute to reducing the incidence of death, while aging and the proportion of the minority population increase the risk of death. The relationship between medical care and mortality was negative. Compared to the distribution in eastern China, which had a higher level of economic and medical development, the impact of the development of medical level on mortality was relatively reduced, and other unfavorable factors like low efficiency and uneven distribution of medical resources allocation became the leading cause of mortality (49, 50).

**Table 4:** GTWR model test results.

	**Average**	**Min**	**25%**	**50%**	**75%**	**Max**	**Standard deviation**
C1_PM_2.5_ (*100)	0.46	−2.85	0.00	0.45	0.90	5.98	0.97
C2_CLS (*100)	0.10	−2.04	−0.41	−0.03	0.21	1.03	0.48
C3_ELD	0.38	0.09	0.36	0.38	0.41	0.60	0.06
C4_EDU	−0.68	−0.86	−0.75	−0.69	−0.64	−0.21	0.09
C5_MED (*100)	0.55	−1.56	0.36	0.60	0.87	1.38	0.51
C6_GDPpc (*100)	−1.01	−8.05	−1.46	−0.57	−0.12	0.23	1.25
C7_PMP (*100)	0.47	−1.72	−0.22	−0.28	1.24	3.35	0.90

**Figure 6:**
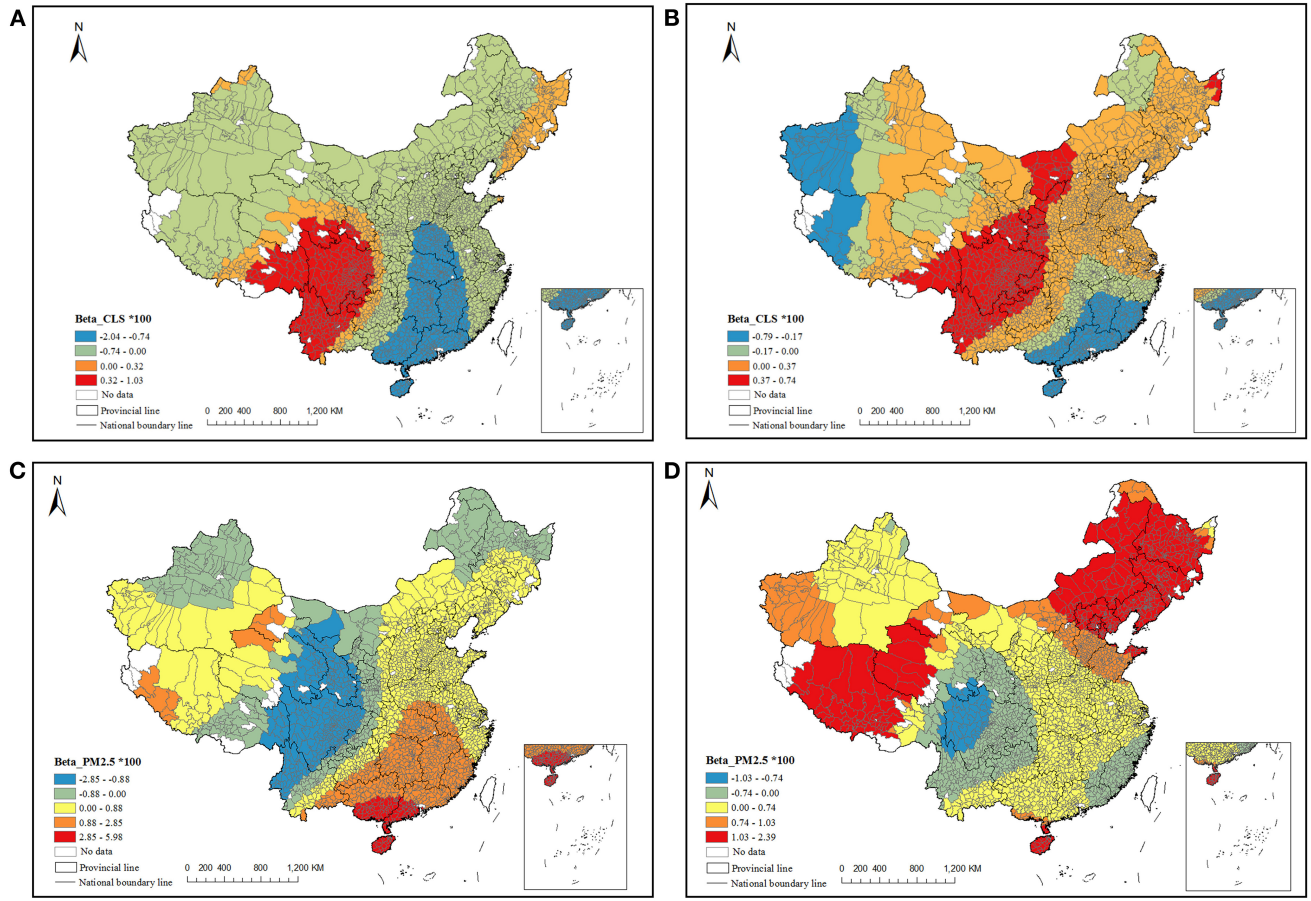
The local non-stationary influence of CLS and PM_2.5_ on mortality **(A, C)** 2000 **(B, D)** 2010.

To identify the spatial heterogeneity of the impact of climate sustainability and air pollution on total mortality, the average annual regression coefficients of CLS and PM_2.5_ in the GTWR model were visualized in Figure 6. For CLS, in addition to the southwest region including Sichuan, eastern Tibet, western Yunnan, southern Gansu, and Qinghai, most of the areas were shown to be negatively correlated with mortality in 2000. The higher the CLS, the lower the mortality, especially in Hainan, Guangdong, and Guangxi. In 2010, only the west-central region and a small part of the northeast and northwest regions had a negative correlation between CLS and mortality, indicating that human suitability for climate had increased, and the contribution of climate change to mortality was weakened. For PM_2.5_, only the west-central region and a small part of the northeast and northwest regions were negatively correlated in 2000, while other regions were positively correlated, meaning the higher the PM_2.5_ concentration, the higher the mortality. In 2010, the areas with a positive relation between PM_2.5_ and mortality had increased, indicating that the increase of PM_2.5_ increased its threat to human health.

## 4. Discussion

This study estimated the influence of air pollution exposure and climate sustainability on total mortality based on county panel data in China. Spatial-temporal heterogeneity of mortality was found, with high-high or low-low spot distribution in China having more regional coherence from 2000 to 2010. The mortality inequity was in general lower within provinces, while higher at the country level, and the inequity showed an increasing trend from 2000 to 2010. GAM was then used to explore the interactive effect of PM_2.5_ and CLS on mortality, and further, GTWR model was used to identify the spatiotemporal factors.

The deviance explained was much higher in the GAM model when considering the interactive effect and an antagonistic effect was found on the mortality. Generally, the higher the PM_2.5_ concentration and the lower the CLS, the higher the rate of mortality, especially when PM_2.5_ concentrations exceeded 35 μg/m^3^ and CLS was below 100, the interactive effect became significant. The climate sustainability indicator here was comprised of temperature, humidity, altitude, and wind-chill index. Too high or too low will cause human maladjustment, and thus the antagonistic effect was less. For example, the cold temperature may increase cardiovascular strain in healthy individuals *via* physiological means to maintain heat balance (51, 52), hot temperature and low relative humidity are associated with increased loss of water from the body through the skin as well as the mucus membrane, while hot temperature and high humidity may trigger asthma symptoms by increasing airway resistance. The process of physiological adjustment of ambient temperature may affect the intake of toxic substances, thereby increasing the total intake of air pollution in the human body. Many reports have confirmed that the combined effect of adverse climate factors and PM_2.5_ would increase the risk of circulatory and respiratory diseases and cardiovascular diseases, as well as the total mortality (14, 15, 28, 53–55).

According to the GAM results, when CLS was in the range of 50–150, the influence on mortality was stable, meaning that this period may be the most comfortable for humans. When below 50, the higher the CLS, the lower the mortality risk. These values are mainly distributed in the plateau areas since human health is affected by a high-altitude hypoxia environment. Exposure to increasing terrestrial altitude reduces ambient O_2_ availability in cells producing a series of hypoxic oxidative stress reactions altering the redox balance in humans (56–59). When CLS was above 150, impact of CLS on mortality showed a positive relationship. The counties with high CLS mainly located in Northeast China and the high wind-chill index contributed to the high CLS, indicating an uncomfortable environment for people to live in. For air pollution, when PM_2.5_ was more than 35 μg/m^3^, the influence on mortality becomes significantly increased as PM_2.5_ concentration increased, and when above 70 μg/m^3^, the trend is sharp. This phenomenon provides some support for the concentration threshold of PM_2.5_ in the standards of China, which stipulated 35 μg/m^3^ as the top and 75 μg/m^3^ as the second level. Though it is not that strict with the air quality guidelines of WHO, especially updated in 2021 (annual PM_2.5_ AQG revised from 10 μg/m^3^ to 5 μg/m^3^) (60). Considering that during this study period the main source of air pollution was coal combustion in China, carbonaceous material and sulfate were the main components for PM_2.5_, which are less toxic compared with the recent components of volatile organic compounds (like Polycyclic Aromatic Hydrocarbons) and heavy metals, from the transportation and chemical industry (61–67). This could be one of the reasons that a more severe mortality influence in 2010 than in 2000 was found. Identically, although the pollution control policy strengthened, the current ambient pollution standard may not satisfy the present-day situation in China, and more stringent standards should be developed.

Regarding spatial-temporal heterogeneity, comparing the results of 2 years with GTWR, we found that counties with a negative relationship between CLS and mortality had decreased, while counties with a positive between PM_2.5_ and mortality had increased, indicating more people are at risk of PM_2.5_ exposure with time went by. Besides the worse air pollution in 2010, the increasing aging may amplify the health risk of PM_2.5_ (68), and the economic and medical improvements may not be enough to offset the serious health threats posed by aging. An abnormal relationship between PM_2.5_ and mortality was found in part of the southwest areas, as well as between CLS and mortality. It may be related to the special geographical conditions within the transition zone from basin to plateau, and there are reports that the climate index was insensitive there, and the weather would change drastically in a year. The regions with positive relationship between CLS and mortality significantly expanded from 2000 to 2010, and the change was more evident in the northeast. There are two possible explanations for this. First, in northeast, the CLS in almost all regions are higher than 150 with high wind-chill index, which might impact the mortality as the similar results from the GAM. While for the rest region, CLS are in the range of 50–150 and the relationship between CLS and mortality is more subtle. Thus, slight human disturbance might affect its variation. In particular, in 2007, government issued China's Policies and Actions to Address Climate Change, the observation and forecast system of climate change was fully established in 2010, and the public awareness to cope with climate change has been strengthened since then. This might lead to the uncertainty between CLS and mortality. In addition, the improved living standards to facilitate the intake of nutrition, the use of air-conditioning, heating and other equipment has further improved people's ability to adapt to climate change in recent years (18, 55). There is one example that there is a high-related mortality risk in areas with lower GDP where air conditioning cannot be afforded (15). Moreover, individuals usually make more substantial adaptation decisions in the end, to alleviate and mitigate health risks due to extreme temperature (69). Another possible explanation for this is that the GTWR model is a linear regression; while the impact of CLS on mortality is not a one-way (positive or negative) correlation.

Our study has several limitations. First, in the calculation method of the CLS, the annual mean value of climate factors such as temperature, humidity, and wind speed are adopted in our study, but extreme events such as extreme temperature are not included. This is a less comprehensive assessment of the impact of climate change as a whole, although it can reflect the sensory perception of climate change for people, to some extent. Second is the source of PM_2.5_ data. Although the interpretation degree used in this study was 81%, the unified air pollution monitoring stations were not set up before 2013 in China, and we could not evaluate the accuracy of the data for the 2 years. Therefore, there could be some errors. Third, we used the total population mortality rate as the dependent variable. The impact of climate change and air pollution on health would also be affected by gender, age and occupation, while this study was based on the population level and not the individual level.

## 5. Conclusions and suggestions

This study explored the spatial-temporal change of mortality, its inequity, and the influencing mechanisms of CLS and PM_2.5_ based on county level penal data in 2000 and 2010. Four main conclusions can be drawn. First, spatial heterogeneity of mortality existed and the mortality inequity decreased with the years. Second, considering the individual effect, when CLS is much higher than 150 or lower than 50, the higher the mortality. When PM_2.5_ was more than 35 μg/m^3^, the influence on mortality became significantly increased especially above 70 μg/m^3^. Third, an antagonistic effect between climate sustainability and air pollution was found. When CLS was lower (below 100) and PM_2.5_ was higher (above 35 μg/m^3^), the antagonistic effect was stronger. It suggests specific health protection attention should be paid in these regions, mainly in northwest and central China. Fourth, from a spatial perspective, the impact of CLS and PM_2.5_ on health varies greatly in different geographical regions. In 2010, the negative health effects of PM_2.5_ are more serious when the PM_2.5_ is lower than 35 μg/m^3^, indicating the necessity to further strengthen the national air quality guidelines in China.

## Data availability statement

The original contributions presented in the study are included in the article/Supplementary material, further inquiries can be directed to the corresponding authors.

## Author contributions

LW, HL, and YG conceptualized this study and did the writing. YG and LW conducted the model analysis. YG, LQ, LY, and LZ collected the data. All authors contributed to the article and approved the submitted version.
