# Supplementary material for: County level study of the interaction effect of PM2.5 and climate sustainability on mortality in China
Source: Front Public Health. 2023 Jan 6;10:1036272. doi: 10.3389/fpubh.2022.1036272 (PMC9853058; doi:10.3389/fpubh.2022.1036272)
Supplement: Supplementary file 1 [file Data_Sheet_1.docx]

**Supplementary information**

**1、The detailed calculation process of CLS**

The temperature-humidity index(THI), wind-chill index(WCI), clothing index(CI), and altitude adaptation index(AAI) were established first of the formula (1), (2), (3) and (4). And then constructed into a comprehensive index with weighted coefficients (formula (5)).

$THI=(1.8t+32)-0.55(1-f)(1.8t-26)$ (1)

$WCI=(33-t)(9.0+10.9\sqrt{V}-V)$ (2)

$CI=\frac{33-t}{0.155H}-\frac{H+\alpha\cos a}{(0.62+19.0\sqrt{V})H}$ (3)

$AAI=(3000-i)/3000$ (4)

$CCI=(0.6\times THI+0.3\times WCI+0.1\times CI)\times AAI$ (5)

Where, t is the temperature (℃), f is the relative humidity (%), V is the wind speed(m/s), H is the 75% of the human metabolic rate, a is the absorption of solar radiation by human body, R is the solar radiation received by the land per vertical unit(W/m^2^), $\alpha$ means the solar elevation angle, and i is the altitude(m). The classification criteria and human sensation of each index are shown in Table S1.

Table S1 Classification criteria of THI, WCI, CI and AAI

| **THI** | | **WCI** | | **CI** | | **AAI** | |
| --- | --- | --- | --- | --- | --- | --- | --- |
| **Classification** | **Human body sensation** | **Classification** | **Human body sensation** | **Classification** | **Suitable clothing** | **Oxygen content of air** | **Human body sensation** |
| < 40 | Extremely cold and uncomfortable | ≤80 | Hot | >2.5 | Down coat or fur coat | 0m，100% | Very comfortable |
| 40 ~ 45 | Cold and uncomfortable | 80 ~ 100 | Warm | 1.8 ~ 2.5 | Casual clothing plus coat | 100m，99.2 | Comfortable |
| 45 ~ 55 | Slightly cold and uncomfortable | 100 ~ 300 | Suitable | 1.5 ~ 1.8 | Casual winter clothing | 1000m，92.4% | Quite comfortable |
| 55 ~ 60 | Cool and comfortable | 300 ~ 500 | Cool | 1.3 ~ 1.5 | Casual spring and autumn clothing | 2000m，84.7% | Generally comfortable |
| 60 ~ 65 | Cool and very comfortable | 500 ~ 700 | Very cool | 0.7 ~ 1.3 | Shirt and casual clothing | 3000m，77.1% | Uncomfortable |
| 65 ~ 70 | Warm and comfortable | 700 - 900 | Cold | 0.5 ~ 0.7 | Light summer clothing | 4000m，69.5% | Very uncomfortable |
| 70 ~ 75 | Rather warm and comfortable | 900 ~ 1100 | Very cold | 0.3 ~ 0.5 | Short sleeve and open collar shirt | 5000m，61.8% | Extremely uncomfortable |
| 75 ~ 80 | Sultry and uncomfortable | 1100 ~ 1300 | Freezing cold | 0.1 ~ 0.3 | Tropical light clothing |  |  |
| > 80 | Extremely sultry and uncomfortable | ≥ 1300 | Unbearable cold | < 0.1 | Extremely light clothing |  |  |

**2、The distribution of total mortality, PM_2.5_ and CLS at county level**

| 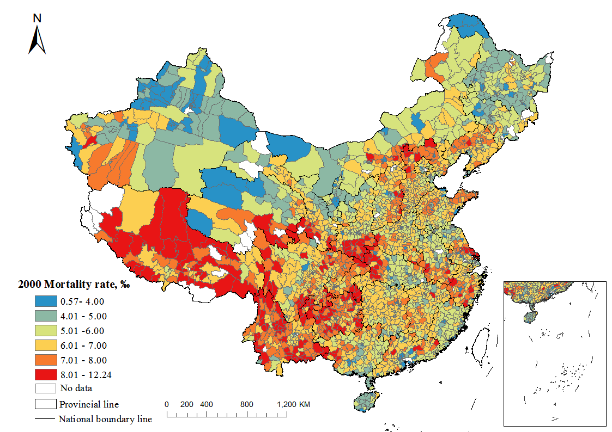 | **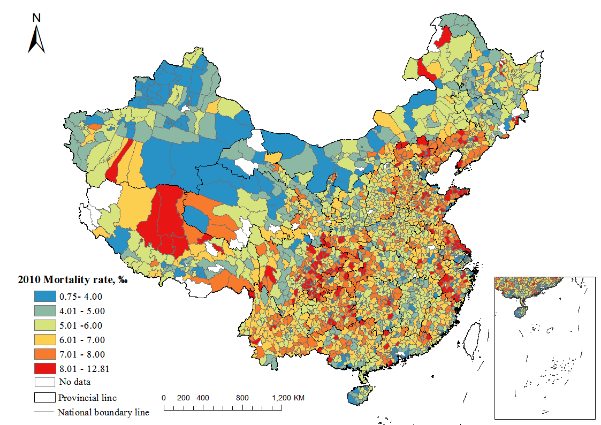** |
| --- | --- |
| **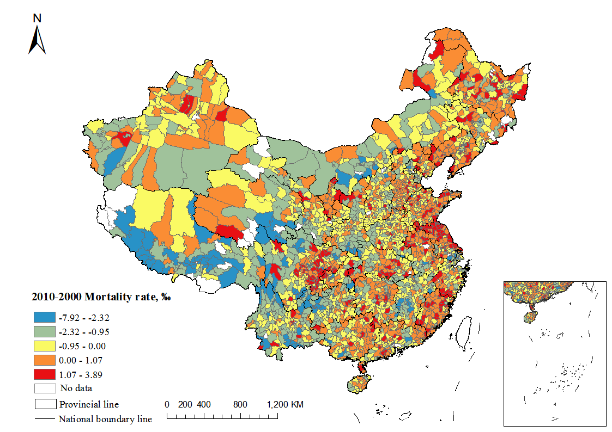** | |

**Figure S1. The county level of total mortality distribution in China**

| 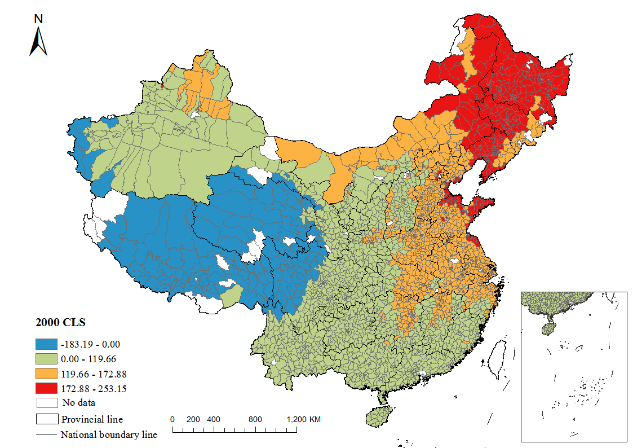 | 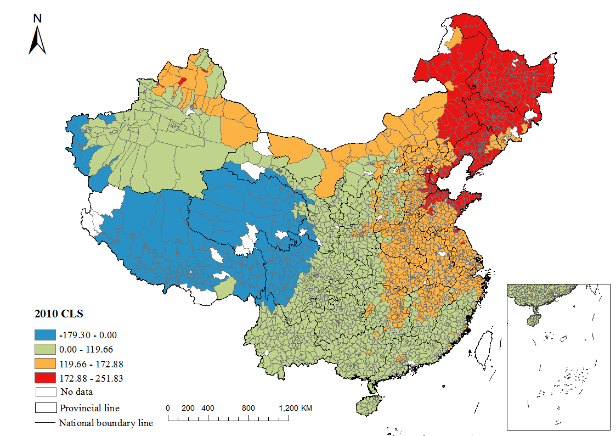 |
| --- | --- |
| 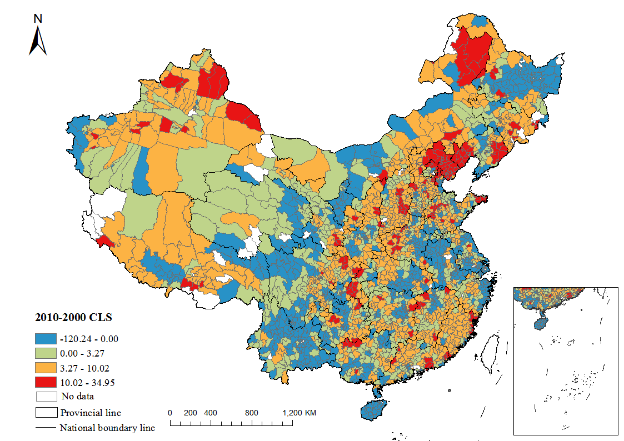 | |

**Figure S2. The county level of CLS distribution in China**

| 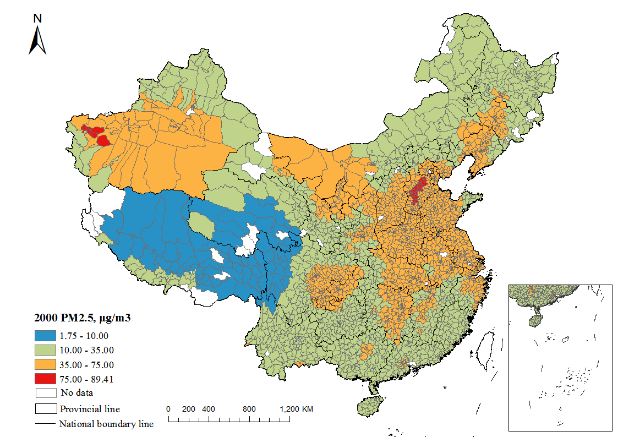 | 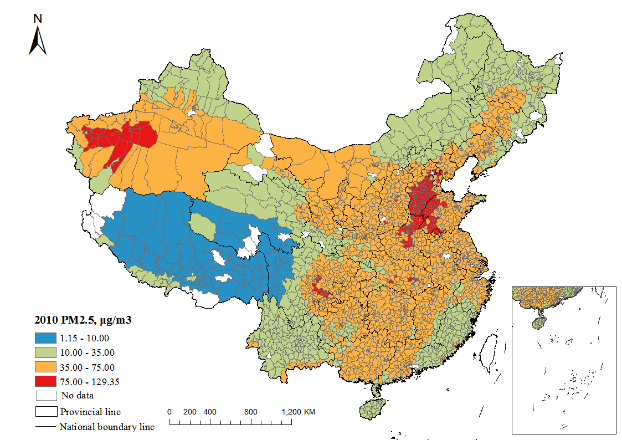 |
| --- | --- |
| 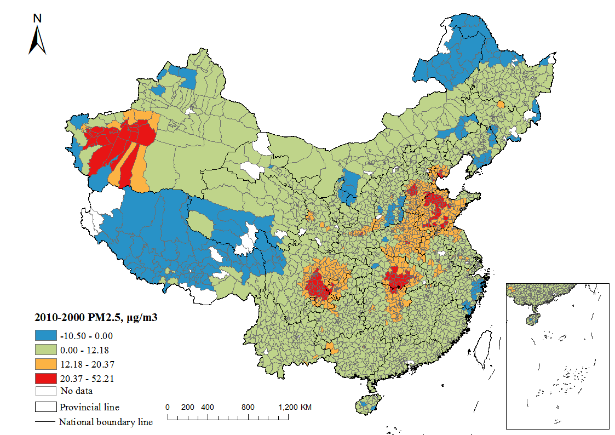 | |

**Figure S3. The county level of PM_2.5_ distribution in China**
